# Polyphosphate uses mTOR, pyrophosphate, and Rho GTPase components to potentiate bacterial survival in *Dictyostelium*

**DOI:** 10.1128/mbio.01939-23

**Published:** 2023-09-27

**Authors:** Ryan J. Rahman, Ramesh Rijal, Shiyu Jing, Te-An Chen, Issam Ismail, Richard H. Gomer

**Affiliations:** 1 Department of Biology, Texas A&M University, College Station, Texas, USA; School of Medicine, Oregon Health & Science University, Portland, Oregon, USA

**Keywords:** polyphosphate, *Dictyostelium*, phagocytosis, bacterial survival

## Abstract

Human macrophages and the eukaryotic microbe *Dictyostelium discoideum* ingest bacteria by phagocytosis, and then kill the ingested bacteria. Some pathogenic bacteria secrete linear chains of phosphate residues (polyphosphate; polyP), and the polyP prevents some of the phagocytes from killing the ingested bacteria. In *D. discoideum,* the effect of polyP requires the G protein-coupled receptor (GPCR) GrlD, suggesting that polyP uses a signal transduction pathway to inhibit killing of ingested bacteria. Here we show that in addition to GrlD, the *D. discoideum* polyP signaling pathway requires the GPCR interacting arrestin-like protein AdcB, inositol hexakisphosphate kinase A (I6kA), the Rho GTPase RacE, and the target of rapamycin (TOR) component Lst8. *D. discoideum* also secretes polyP, and at high concentrations polyP inhibits *D. discoideum* cytokinesis. The polyP inhibition of bacterial killing pathway has some components that overlap and some components that are distinct from the polyP inhibition of cytokinesis pathway. These data suggest the intriguing possibility that if there is a similar polyP inhibition of bacterial killing pathway in macrophages, pharmacologically blocking this pathway could potentiate macrophage killing of pathogenic bacteria.

**IMPORTANCE:**

Although most bacteria are quickly killed after phagocytosis by a eukaryotic cell, some pathogenic bacteria escape death after phagocytosis. Pathogenic *Mycobacterium* species secrete polyP, and the polyP is necessary for the bacteria to prevent their killing after phagocytosis. Conversely, exogenous polyP prevents the killing of ingested bacteria that are normally killed after phagocytosis by human macrophages and the eukaryotic microbe *Dictyostelium discoideum*. This suggests the possibility that in these cells, a signal transduction pathway is used to sense polyP and prevent killing of ingested bacteria. In this report, we identify key components of the polyP signal transduction pathway in *D. discoideum*. In cells lacking these components, polyP is unable to inhibit killing of ingested bacteria. The pathway components have orthologs in human cells, and an exciting possibility is that pharmacologically blocking this pathway in human macrophages would cause them to kill ingested pathogens such as *Mycobacterium tuberculosis*.

## INTRODUCTION

Polyphosphate (polyP) is a linear polymer of three to hundreds of orthophosphate residues, and is found in all kingdoms of life (1). PolyP is predominantly synthesized by polyphosphate kinase 1 (Ppk1), which is highly conserved in more than 100 prokaryotes, including more than 20 major bacterial pathogens and a few eukaryotes such as *Dictyostelium discoideum* (2). Bacteria lacking Ppk1 lose essential functions for survival, such as cell motility, biofilm formation, and pathogenicity (3 – 5). The pivotal role polyP plays in pathogenesis has marked Ppk1, which is absent in humans, as a potential target to block pathogenicity (6).

In mammalian cells, polyP (*n* = ~50–150 phosphates) is concentrated in platelet and mast cell granules and is released during injury or cell activation to potentiate blood clotting cascades (7). Platelet-released polyP also triggers release of neutrophil extracellular traps (8), induces macrophage differentiation and neutrophil chemoattraction during wound healing (9), but inhibits peritoneal macrophage chemotaxis to the sites of infection and tissue damage (10). PolyP can also signal through receptors such as P2Y1 and RAGE to potentiate pro-inflammatory responses of endothelial cells as well as mediate communication among astrocytes (11, 12).

Macrophages fight bacterial infections by phagocytosis (13). During phagocytosis, a bacterium is engulfed into a phagosome, which then acidifies and fuses with a lysosome to form a phagolysosome to kill and digest the ingested bacterium (14). Pathogens such as *Mycobacterium tuberculosis* (*Mtb*) prevent their killing in human macrophages by inhibiting phagosome acidification and fusion of the phagosome with the lysosome (15). We previously observed that *Mycobacterium smegmatis* and *Mtb* secrete extracellular polyP (16), that exogenous polyP inhibits phagosome acidification and lysosome activity, and that polyP potentiates the survival of non-pathogenic *Escherichia coli* in human macrophages (16). Other workers found that exogenous polyP potentiates pathogenic *E. coli* survival in a murine model of sepsis (17). Conversely, treatment of human macrophage and *Mtb* co-cultures with the polyP-degrading enzyme exopolyphosphatase (PPX), or reduced expression of Ppk1 in *M. smegmatis*, reduced the survival of these bacteria in human macrophages (16, 18). Together, these results suggest that extracellular polyP produced by some pathogenic bacteria contributes to their survival in macrophages (16).

*D. discoideum* is a eukaryotic microbe that primarily feeds on bacteria by phagocytosis (19 – 21). Many *D. discoideum* proteins involved in phagocytosis are conserved in human neutrophils and macrophages (22). Proliferating *D. discoideum* cells accumulate extracellular polyP, and as the cell density increases to near the point where the cells are about to overgrow their food supply and starve, the concomitant high levels of extracellular polyP inhibit cytokinesis but not the accumulation of cell mass so that the cells will be large and have high nutrient reserves when they starve (23). The extracellular polyP is sensed by the putative G protein-coupled polyP receptor GrlD (24). PolyP inhibits proliferation through distinct mechanisms based on nutrient availability, as GrlD partially mediates this effect in high-nutrient conditions while GrlD and a small GTPase RasC are necessary for the effect in low-nutrient conditions (24). In addition to GrlD and RasC in low-nutrient conditions, polyP requires the G protein component Gβ, the Ras guanine nucleotide exchange factor GefA, phosphatase and tensin homolog (PTEN), phospholipase C (PLC), inositol 1,4,5-trisphosphate (IP3) receptor-like protein A (IplA), Ppk1, and the TOR complex two component PiaA to inhibit proliferation (25). With the exception of *grlD^−^*, *rasC^−^*, and *piaA^−^*, the strains lacking the aforementioned proteins had reduced but non-zero responses to polyP, suggesting the existence of parallel pathways mediating polyP effects (25).

In *D. discoideum,* polyP acts via the polyP receptor GrlD to potentiate *E. coli* survival (16). We previously observed that *E. coli*, which do not accumulate detectable extracellular polyP, get killed after phagocytosis by *D. discoideum*, while *M. smegmatis* that accumulate detectable extracellular polyP survive better after phagocytosis than *E. coli* (16). As in macrophages, reduced expression of *ppk1* in *M. smegmatis* bacteria reduces their survival in *D. discoideum,* and the addition of exogenous polyP potentiates their survival (16). Together, this suggests the intriguing possibility that there is a signal transduction pathway whereby either extracellular polyP or polyP secreted by a bacterium in a phagosome prevents cells from fusing the phagosome with a lysosome. In this report, we screened *D. discoideum* mutants to elucidate polyP signal transduction pathways that are needed for polyP to potentiate the survival of ingested bacteria in *D. discoideum*. We find that extracellular polyP requires the G protein-coupled receptor (GPCR) interacting arrestin-like protein AdcB, inositol hexakisphosphate kinase A (I6kA), the Rho GTPase RacE, and the TOR component Lst8 to potentiate *E. coli* survival in *D. discoideum*.

## RESULTS

### PolyP requires a G protein-coupled receptor but does not require G-protein subunits to potentiate the survival of *Escherichia coli* in *D. discoideum*

**Fig 1:**
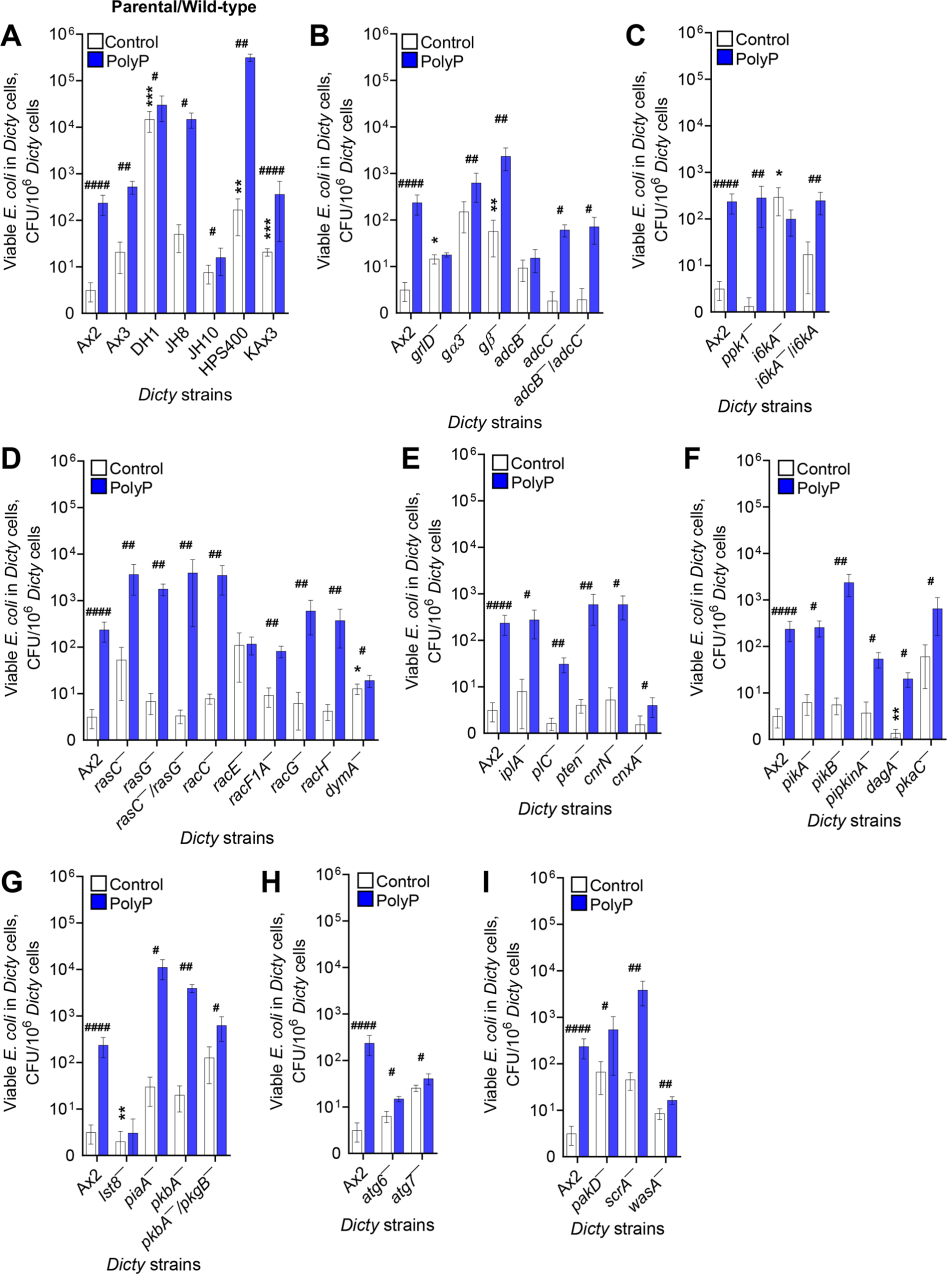
PolyP potentiates the long-term survival of ingested *E. coli* in D. discoideum. (**A–I**) D. discoideum (Dicty) cells were incubated with *E. coli*, uningested *E. coli* were removed, and the number of viable ingested *E. coli* per 10^6^ *D. discoideum* cells in the absence (Control) or the presence of added polyphosphate (PolyP) was determined at 48 hours. Values are mean ± SEM from five independent experiments for each mutant/parental strain and 16 independent experiments for Ax2 wild type. *P < 0.05, **P < 0.01, and ***P < 0.001 by two-tailed Mann-Whitney test comparing the indicated mutant to its parental strain (Table 1), or the indicated parental strain to Ax2, in the absence of added polyP. #*P* < 0.05, ##*P* < 0.01, and ####*P* < 0.0001 by two-tailed Mann-Whitney test comparing control to polyP for the indicated strain.

**Fig 2:**
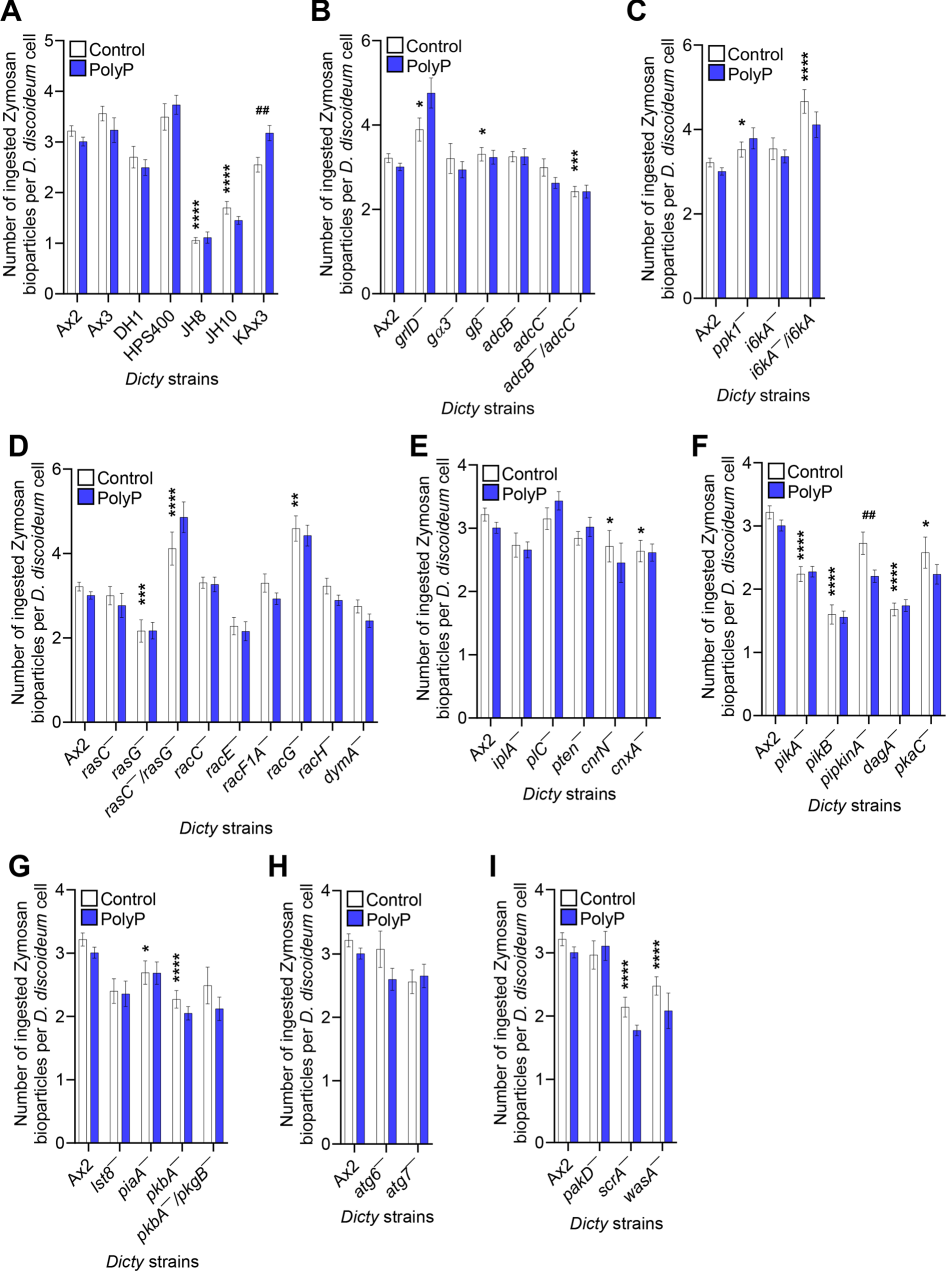
PolyP has negligible effect on the uptake of Zymosan A bioparticles in most strains. (**A-I**) The number of Zymosan A bioparticles ingested per *D. discoideum* cell in 60 minutes in the presence or absence of 15 µg/mL polyP was determined. Values are mean ± SEM from three independent experiments. *P < 0.05, **P < 0.01, ***P < 0.001, and ****P < 0.0001 comparing the indicated mutant to its parental strain, or the indicated parental strain to Ax2, in the absence of added polyP by two-tailed Mann-Whitney test. ##*P* < 0.01 comparing control and polyP for the indicated strain by two-tailed Mann-Whitney test.

Wild-type (WT) *D. discoideum* cells accumulate extracellular polyP, and the polyP concentrations (≥470 µg/mL) corresponding to high cell densities (≥1 × 10^7^ cells/mL) inhibit proliferation, macropinocytosis, exocytosis, and killing of ingested bacteria (23, 26). PolyP concentrations between 5 and 47 µg/mL, which do not affect proliferation, macropinocytosis, or exocytosis, inhibit the killing of ingested bacteria in *D. discoideum* (16). To identify components of the polyP signal transduction pathway that potentiate the survival of bacteria in *D. discoideum*, 37 available mutants, representing a wide variety of signal transduction pathways and processes, derived from seven parental strains were tested for sensitivity to polyP-mediated *E. coli* survival as previously described (16) using exogenous polyP as the source of polyP since *E. coli* do not accumulate detectable levels of extracellular polyP (16) (Table 1). In these assays, *Dictyostelium* cells are allowed to ingest *E. coli* bacteria, the uningested bacteria are washed off, any remaining uningested bacteria are killed with the antibiotic gentamicin, which does not kill ingested bacteria (27), and at 4 and 48 hours, aliquots of the *Dictyostelium* cells are counted and are lysed with a detergent that does not kill *E. coli,* and the ingested bacteria are plated to obtain a count of live bacteria. Adding extracellular polyP has little effect on the bacterial survival at 4 hours, and strongly potentiates survival at 48 hours (16). To determine if polyP-mediated changes in intracellular *E. coli* numbers correspond to altered ingestion or digestion, the efficiency of phagocytic engulfment of Zymosan A bioparticles was measured for all strains. The data are graphed in nine groups: parental/wild-type cells (Fig. 1A and 2A; Fig. S1A and S2A), polyP receptor, G-proteins, and arrestin-like proteins (Fig. 1B and 2B; Fig. S1B and S2B), proteins involved in polyP production (Fig. 1C and 2C; Fig S1C and S2C), GTPases (Fig. 1D and 2D; Fig. S1D and S2D), phospholipase C (PLC)/IP3 pathway components (Fig. 1E and 2E; Fig. S1E and S2E), PI3 kinase signal transduction pathway components (Fig. 1F and 2F; Fig. S1F and S2F), TOR complex components/protein kinases (Fig. 1G and 2G; Fig. S1G and S2G), autophagy pathway components (Fig. 1H and 2H; Fig. S1H and S2H), and cytoskeleton regulating proteins (Fig. 1I and 2I; Fig. S1I and S2I).

**TABLE 1:** *D. discoideum* sensitivity to polyP^*a*^

Strain	Parental strain	Ingestion (phagocytic index) relative to the respective parental strain (Fig. 2)	Viability of ingested *E. coli* at 4 hours relative to the respective parental strain (Fig. S1)	Viability of ingested *E. coli* at 48 hours relative to the respective parental strain (Fig. 1)	Sensitivity to polyP potentiation of viability of ingested *E. coli* at 48 hours (Fig. 1)
**Ax2**		Normal	Normal	Normal	Sensitive
**Ax3**		Normal	Normal	Normal	Sensitive
**DH1**		Normal	More	More	Sensitive
**HPS400**		Normal	More	More	Sensitive
**JH8**		Low	Normal	Normal	Sensitive
**JH10**		Low	Normal	Normal	Sensitive
**KAx3**		Normal	Normal	More	Sensitive
***adcB^¯^***	**Ax2**	**Same**	**Same**	**Same**	**Insensitive**
***adcB^¯^/adcC^¯^***	Ax2	Less	Same	Same	Sensitive
***adcC^¯^***	Ax2	Same	Same	Same	Sensitive
***atg6^¯^***	Ax2	Same	Same	Same	Sensitive
***atg7^¯^***	Ax2	Same	More	Same	Sensitive
***cnrN^¯^***	Ax2	Less	Same	Same	Sensitive
***cnxA^¯^***	Ax2	Less	Same	Same	Sensitive
***dagA^¯^***	Ax3	Less	Less	Less	Sensitive
***dymA^¯^***	Ax2	Same	More	More	Sensitive
***grlD^¯^***	**Ax2**	**More**	**More**	**More**	**Insensitive**
***gα3^¯^***	HPS400	Same	Same	Same	Sensitive
***gβ^¯^***	DH1	More	Less	Less	Sensitive
***i6kA^¯^***	**Ax2**	**Same**	**Same**	**More**	**Insensitive**
***i6kA^¯^/i6kA***	Ax2	More	Same	Same	Sensitive
***iplA^¯^***	Ax2	Same	Same	Same	Sensitive
***lst8^¯^***	**KAx3**	**Same**	**Less**	**Less**	**Insensitive**
***pakD^¯^***	Ax2	Same	More	Same	Sensitive
***piaA^¯^***	Ax2	Less	Same	Same	Sensitive
***pikA^¯^***	Ax2	Less	Same	Same	Sensitive
***pikB^¯^***	Ax2	Less	Same	Same	Sensitive
***pipkinA^¯^***	Ax2	Same	Same	Same	Sensitive
***pkaC^¯^***	JH10	More	Same	Same	Sensitive
***pkbA^¯^***	Ax2	Less	Same	Same	Sensitive
***pkbA^¯^/pkgB^¯^***	Ax2	Same	Same	Same	Sensitive
***plC^¯^***	Ax2	Same	Same	Same	Sensitive
***ppk1^¯^***	Ax2	More	Same	Same	Sensitive
***pten^¯^***	Ax2	Same	More	Same	Sensitive
***racC^¯^***	Ax2	Same	Same	Same	Sensitive
***racE^¯^***	**DH1**	**Same**	**Same**	**Same**	**Insensitive**
***racF1A^¯^***	Ax2	Same	Same	Same	Sensitive
***racG^¯^***	Ax2	More	Same	Same	Sensitive
***racH^¯^***	Ax2	Same	Same	Same	Sensitive
***rasC^¯^***	Ax2	Same	Same	Same	Sensitive
***rasC^¯^/rasG^¯^***	Ax2	More	More	Same	Sensitive
***rasG^¯^***	Ax2	Less	Same	Same	Sensitive
***scrA^¯^***	JH8	More	Same	Same	Sensitive
***wasA^¯^***	Ax3	Less	Less	Same	Sensitive

^*a*^ Forty-four *D*. *discoideum* strains including seven parental wild-type strains were tested for sensitivity to polyP for ingestion of Zymosan bioparticles and viability of ingested *E. coli* at 4 and 48 hours. Parental strains were compared against Ax2, which was considered normal, and mutants were compared against their parental strains and considered less, same, or more for the indicated parameters tested. Mutants that responded or did not respond to polyP potentiation of viability of ingested bacteria at 48 hours were considered sensitive or insensitive (highlighted in bold), respectively.

At 4 hours, polyP did not affect the number of viable ingested *E. coli* in the parental/WT strains or any of the mutants, with the exception of cells lacking the arrestin-like protein AdcC, where polyP caused a slight drop in the number of viable internalized *E. coli* (Fig. S1A to I; Table 1). At 48 hours, although DH1, HPS400, and KAx3 parental strains had significantly increased numbers of viable ingested *E. coli* in the absence of exogenous polyP compared to the Ax2 parental strain, polyP increased the number of viable, ingested *E. coli* in all of the parental strains (Fig. 1A and Table 1). In addition to the differences (in the absence of exogenous polyP) between these parental strains in the number of viable bacteria at 4 and at 48 hours (Fig. 1A; Fig. S1A; Table 1), there were also differences in phagocytosis (Fig. 2A; Fig. S2A; Table 1), indicating some strain-dependent differences in phagocytosis and phagosome/lysosome physiology. This may be due to genetic variations between different parental axenic strains (28). Some of the parental strains are auxotrophs, which may also affect phagocytosis and lysosomes.

To test if polyP affects *Dictyostelium* viability when incubated with bacteria, *Dictyostelium* cells with ingested bacteria at 4 and 48 hours of phagocytosis were stained with Trypan blue and the live and dead cells were counted. At 4 hours, there was no difference in *Dictyostelium* total, live, and dead cell densities in the absence or presence of polyP. At 48 hours, the total and live cell densities increased approximately threefold, and the presence of polyP did not affect the total, live, and dead cell densities (Fig. S3A), suggesting that polyP reduces the ability of *D. discoideum* cells to kill the ingested bacteria without affecting the viability of *Dictyostelium* cells.

Cells lacking the putative G protein-coupled polyP receptor GrlD are insensitive to polyP-induced proliferation inhibition (24, 25). Cells lacking the heterotrimeric G protein subunits Gβ and Gα3 have a reduced but non-zero sensitivity to polyP (25), suggesting the existence of an additional pathway downstream of GrlD. Compared to their Ax2 parental strain in the absence of exogenous polyP, cells lacking GrlD had slightly higher phagocytosis and higher viability of ingested bacteria at 4 and 48 hours (Fig. 1A, B and 2A, B; Fig. S1A and B; Table 1). GrlD was needed for polyP to potentiate *E. coli* viability at 48 hours (Fig. 1B and Table 1). These results suggest that GrlD inhibits phagocytosis and mediates the ability of polyP to potentiate the viability of ingested bacteria.

Compared to their HPS400 parental strain, cells lacking Gα3 had normal phagocytosis and viability of ingested *E. coli* (Fig. 1A, B and 2A, B; Fig. S1A and B; Table 1). In response to polyP, cells lacking Gα3 showed increased viability of ingested bacteria, although not as much as parental cells (Fig. 1A and B and Table 1). Compared to their DH1 parental strain, cells lacking Gβ had more phagocytosis, but less viability of ingested *E. coli* at 4 and 48 hours (Fig. 1A, B and 2A, B; Fig. S1A and B; Table 1). In response to polyP, cells lacking Gβ showed increased viability of ingested bacteria (Fig. 1A and B and Table 1). Together, these data suggest that Gβ inhibits phagocytosis and promotes the viability of ingested bacteria, and that polyP uses GrlD to activate a downstream pathway that bypasses, or partially bypasses G proteins.

### PolyP requires the arrestin domain containing protein AdcB to potentiate *E. coli* survival

Persistent activation of GPCRs is dampened by phosphorylation of the cytoplasmic region of the receptors (29). Arrestins are scaffolding proteins that bind to the carboxyl terminus of phosphorylated GPCRs, uncouple GPCRs from their cognate G proteins, and turn off G protein mediated signaling (30, 31). In addition to desensitizing GPCRs, arrestins can also function as adaptor proteins for GPCR trafficking and G protein-independent signaling (32, 33). *D. discoideum* does not possess true arrestins, but has genes encoding six arrestin domain containing proteins (AdcA-F) (34). AdcB but not AdcC was needed for polyP potentiation of bacterial survival at 48 hours, although polyP potentiated bacterial survival in cells lacking both arrestin-like proteins (Fig. 1B and Table 1), suggesting the possibility that AdcB mediates polyP signaling downstream of the GrlD receptor, and that lack of both AdcB and AdcC might cause upregulation of a yet unknown component, possibly one of the four other arrestin-like proteins, that can compensate for the lack of AdcB.

### PolyP requires an inositol hexakisphosphate kinase to potentiate *E. coli* survival

Ppk1 synthesizes polyP from ATP (2, 35). *D. discoideum* cells lacking Ppk1 (*ppk1^−^*) possess undetectable levels of intracellular polyP (36) and reduced but detectable levels of extracellular polyP (23). Inositol hexakisphosphate kinase (IP6K) synthesizes inositol pyrophosphates IP7 and IP8 from IP6 (37). *D. discoideum* cells lacking the IP6K homolog I6kA accumulate reduced levels of extracellular polyP (23). At 48 hours, polyP increased the number of viable ingested *E. coli* in *ppk1^−^* cells but not *i6kA^−^* cells, and the latter defect was rescued in *i6kA^−^/i6kA* cells (Fig. 1C and Table 1), indicating that *D. discoideum* requires I6kA to mediate polyP induced survival of ingested *E. coli*.

### PolyP requires the Rho GTPase RacE to potentiate *E. coli* survival

Ras and Rho GTPases are involved in a variety of cellular processes including proliferation, differentiation, cell motility, cell polarity, and trafficking of vesicles and macromolecules (38, 39). PolyP requires RasC to inhibit proliferation and induce development of *D. discoideum* cells (25, 40), whereas the Rho GTPase RacC was not necessary for polyP mediated proliferation inhibition (25). The ability of polyP to potentiate the survival of ingested bacteria at 48 hours did not require RasC, RasG, RacC, RacF1A, RacG, RacH, or the large GTPase dynamin, which is involved in membrane remodeling during endocytosis and phagocytosis (41, 42) (Fig. 1D and Table 1). Although polyP potentiated the survival of bacteria in cells lacking dynamin (*dymA^−^*), the polyP effect was subtle in *dymA^−^* cells compared to their parental Ax2 cells; this may have some mechanistic connection to the fact that *dymA^−^* cells have inherently more viable, ingested bacteria at 4 and 48 hours (Fig. 1D and Table 1). However, the polyP effect did require the Rho GTPase RacE (43) (Fig. 1D and Table 1).

### PolyP does not require several PLC/IP3 pathway components to potentiate *E. coli* survival

Phospholipase C, which converts PIP2 to diacylglycerol and inositol 1,4,5-trisphosphate (IP3), and the IP3 receptor-like protein IplA are required for polyP-mediated *D. discoideum* proliferation inhibition (25, 44). PTEN and the PTEN-like phosphatase CnrN catalyze the conversion of PIP3 to PIP2 (45, 46) and are involved in many cellular processes including proliferation and cell migration (25, 45, 46). PTEN but not CnrN is involved in polyP-mediated *D. discoideum* proliferation inhibition (25). Calnexin (Cnx) is a calcium binding protein and interacts with IP3 receptors (47). Other potential components of PIP3-associated pathways include phosphatidylinositol kinases PikA and PikB, phosphatidylinositol phosphate kinase A (*pipkinA¯*), the pleckstrin homology (PH) domain containing and PIP3 binding cytosolic regulator of adenylyl cyclase protein CRAC (DagA), and the cAMP-dependent protein kinase A catalytic subunit PkaC. PolyP potentiated bacterial survival at 48 hours in *iplA^−^*, *plC^−^*, *pten^−^*, *cnrN^−^*, *cnxA^−^*, *pikA^−^, pikB^−^, pipkinA^−^ dagA^−^,* and *pkaC^−^* cells (Fig. 1E and F and Table 1), suggesting that polyP does not use these components of the PLC/IP3 pathway to potentiate the survival of ingested bacteria.

### PolyP requires the TOR complex protein Lst8 to potentiate *E. coli* survival

The TOR forms two distinct signaling complexes, TOR complex 1 (TORC1) and TORC2 (48). In mammals, TORC1 activation promotes anabolic metabolism and blocks catabolic processes such as autophagy and lysosome biogenesis (49, 50). TORC2 is involved in cytoskeletal reorganization during chemotactic cell movement (51 – 53). TORC1 and TORC2 complexes have shared and unique components. TOR and Lst8 are present in both TORC1 and TORC2 complexes, whereas PiaA (mammalian Rictor) is unique to TORC2 (48). PolyP requires PiaA to inhibit proliferation of *D. discoideum* cells, whereas cells lacking Lst8 (*lst8^−^*) are sensitive to polyP mediated proliferation inhibition (25). TORC2 regulates the activity of protein kinase B (PKB) (54). PolyP required Lst8 but not PiaA, the Akt/PKB protein kinase PkbA, or PkbA and the SGK family protein kinase PkgB to potentiate bacterial survival (Fig. 1G and Table 1). Compared to their KAx3 parental cells, in the absence of exogenous polyP, *lst8^−^* cells had normal total phagocytosis but an inherently decreased percent of cells showing phagocytosis (Fig. 2A and G; Fig. S2A and G), and fewer viable ingested bacteria at 4 and 48 hours (Fig. 1A and G; Fig. S1A and G; Table 1). Together, this suggests that Lst8 increases the percent of cells that show phagocytosis, increases viability of ingested bacteria, and mediates the ability of polyP to increase the survival of ingested bacteria.

### PolyP does not require the autophagy proteins Atg6 and Atg7 to potentiate *E. coli* survival

Autophagy is used to degrade and recycle cytoplasmic materials in eukaryotic cells (55), and TOR signaling regulates autophagy (56). *D. discoideum* cells feed on bacteria to acquire nutrients in the natural environment. The cells use autophagy machinery to kill bacteria only when the bacteria escape the phagosome in a process called xenophagy (57). *D. discoideum* uses the autophagy proteins Atg6 and Atg7 for autophagosome formation (58). PolyP potentiated the survival of ingested bacteria in *atg6^−^* and *atg7^−^* cells; however, the effect of polyP on *atg6^−^* and *atg7^−^* was mild compared to parental Ax2 cells. (Fig. 1H and Table 1). Together, these data suggest that polyP does not absolutely require Atg6 and Atg7 to potentiate bacterial survival, although these components of the autophagy pathway may contribute to this effect of polyP.

### PolyP does not require selected cytoskeletal proteins to potentiate *E. coli* survival

Actin and actin-associated proteins play critical roles during phagocytic uptake and early phagosome formation processes (59 – 61). Although polyP concentrations (705 µg/mL) corresponding to very high cell densities reduced levels of actin cytoskeleton proteins (40), the lower polyP concentration that potentiates bacteria survival in WT cells did not require the cytoskeleton-associated proteins p21-activated kinase D (PakD), or Wiskott-Aldrich syndrome protein family protein SCAR to potentiate bacterial survival (Fig. 1I), suggesting that polyP prevents the killing of ingested bacteria without requiring these proteins. Although polyP potentiated the survival of bacteria in cells lacking Wiskott-Aldrich syndrome protein (WasA), the effect of polyP was subtle compared to parental Ax3 cells (Fig. 1A and I); this may have a mechanistic connection to, in the absence of exogenous polyP, the reduced phagocytosis, percent of cells showing phagocytosis, and viability of *E. coli* at 4 hours observed in *wasA^−^* cells (Fig. S1A and I; Fig. 2A and I).

### Defective polyP sensitivity is not due to a defect in the parental strain or defective phagocytosis

Cells lacking GrlD, AdcB, I6kA, RacE, and Lst8 (with genotypes verified by PCR, Fig. S3B t0 E) do not potentiate bacterial survival at 48 hours in response to polyP. Ax2 is the parental strain of *grlD^−^*, *adcB^−^*, and *i6kA^−^*, DH1 is the parental strain of *racE^−^*, and KAx3 is the parental strain of *lst8^−^* (Table 1). All of these parental strains increased bacterial survival in response to polyP (Fig. 1A and Table 1), indicating that the defects in the above mutants are not due to a defect in the parental strain. Phagocytosis, as measured by the number of ingested zymosan particles, was normal in most of the above strains with defective polyP responses, with the exception of *grlD^−^*, which had somewhat higher phagocytosis (Fig. 2 and Table 1). Compared to the respective parental strains, there was no consistent effect of these mutations on the percent of cells ingesting beads. Cells lacking GrlD were normal, a slightly higher percentage of cells lacking AdcB and I6kA ingested beads, and a somewhat lower percentage of cells lacking RacE and Lst8 ingested beads (Fig. S2). PolyP had no significant effect on these percentages (Fig. S2). Together, these results indicate that the above proteins are part of a mechanism where extracellular polyP potentiates the survival of ingested bacteria after phagocytosis of the bacteria.

### PolyP inhibits proteasome activity

**Fig 3:**
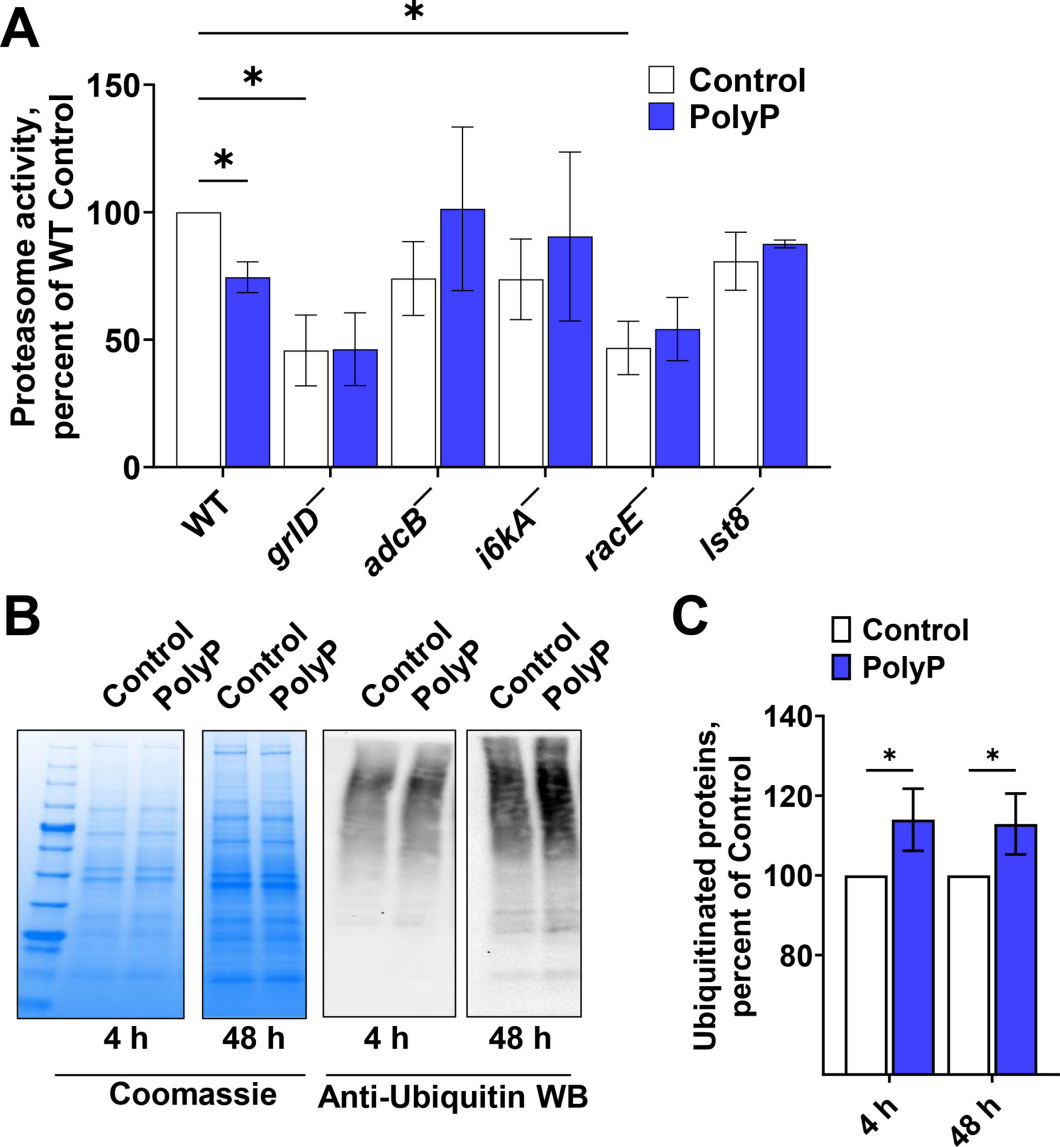
PolyP affects protein ubiquitination and proteasome activity. (**A**) Proteasome activity of Ax2 (WT) and the indicated strains in the presence or absence of 15 µg/mL polyP were measured and normalized to the Ax2 (no polyP) control. Values are mean ± SEM from at least three independent experiments. *P < 0.05 by Holm-Šídák’s multiple comparisons test (two-way ANOVA). (**B**) Coomassie stains and anti-ubiquitin western blot images for 4 and 48 hours Ax2 lysates in the presence and absence of polyP. Images are representative of seven independent experiments. (**C**) The integrated staining intensities in the anti-ubiquitin Western blots were quantified at 4 hours as a percent of the 4 hours control and at 48 hours as a percent of the 48 hours control. Values are mean ± SEM from seven independent experiments. *P < 0.05 by two-tailed Mann-Whitney test.

We previously found that 705 µg/mL polyP (the extracellular polyP concentration in stationary phase cultures) inhibits proteasome activity in *D. discoideum* (40). PolyP requires GrlD and RasC to inhibit proteasome activity in all nutrient conditions (40), but to inhibit proliferation, polyP inhibits proteasome activation only in low nutrient conditions, indicating that polyP inhibits proliferation of *D. discoideum* using different pathways depending on the nutritional conditions (40). To determine if the relatively low concentrations of polyP that potentiate the survival of *E. coli* also inhibit proteasome activity, we tested the effect of 15 µg/mL polyP on proteasome activity in Ax2 and the mutants that are insensitive to polyP-induced bacterial survival. PolyP reduced proteasome activity in Ax2 cells, and although *grlD^−^* and *racE^−^* cells had reduced basal proteasomal activity, polyP did not significantly affect proteasome activity in *grlD^−^, adcB^−^, i6kA^−^, racE^−^,* or *lst8^−^* cells (Fig. 3A). This indicates that GrlD, AdcB, I6kA, RacE, and Lst8 may be parts of a polyP signaling pathway that reduces proteasome activity, although whether this is associated with, or independent of, the effects of this same pathway on survival of ingested bacteria remains unknown.

Cells label proteins with ubiquitin to induce their degradation (62). To determine if polyP potentiation of bacterial survival grossly affects ubiquitinated protein levels, Western blots of *D. discoideum* lysates from the 4 and 48 hours bacterial survival assays were stained with anti-ubiquitin antibodies. PolyP slightly increased the level of ubiquitinated proteins at 4 and 48 hours compared to control (Fig. 3B and C). Together, these data suggest that polyP may modestly increase ubiquitinated protein levels, and that this effect of polyP may be due to reduced proteasome activity, which may cause accumulation of ubiquitinated proteins destined for degradation, and thus may be independent of the effect of polyP on facilitating the long term survival of bacteria.

### PolyP requires AdcB and Lst8 to inhibit cell proliferation

**Fig 4:**
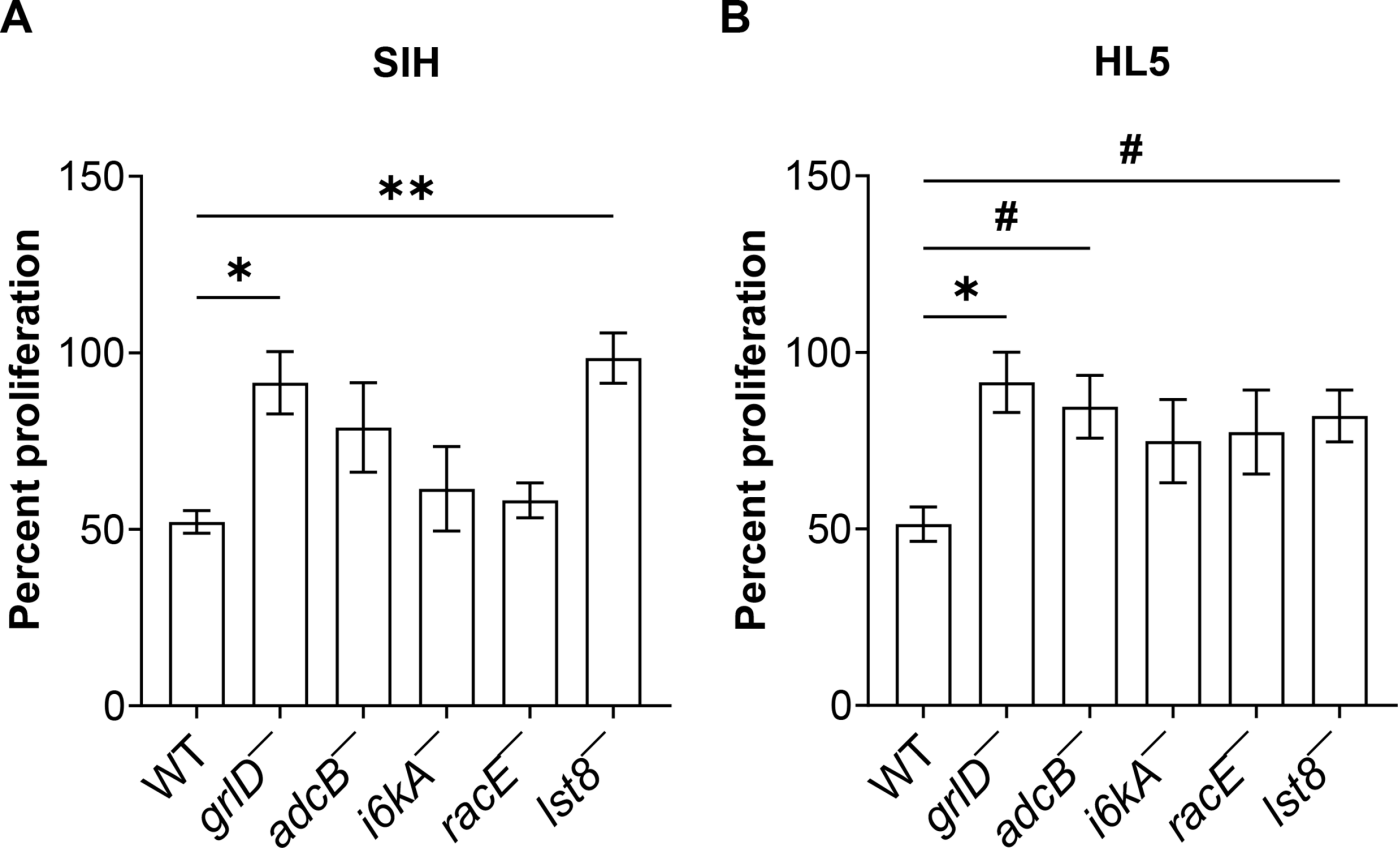
PolyP requires GrlD, AdcB, and Lst8 to inhibit the proliferation of *D. discoideum* cells. Cells were cultured in either SIH (**A**) or HL5 (**B**) with or without 705 µg/mL polyP for 24 hours. The increase in cell density in the presence of polyP was calculated as a percentage of the increase in cell density in the absence of polyP for each strain. Values are mean ± SEM from at least three independent experiments. *P < 0.05 and **P < 0.01 by Holm-Šídák’s multiple comparisons test (one-way ANOVA), and #*P* < 0.05 by two-tailed Mann-Whitney test.

We previously observed that 705 µg/mL polyP inhibits the proliferation of WT *D. discoideum* cells (23), and that the loss of GrlD, I6kA, or TORC2 complex protein PiaA reduces the ability of polyP to inhibit proliferation in a low nutrient (25% HL5) medium (25). To determine if the proteins that mediate polyP potentiation of bacterial survival also affect polyP inhibition of proliferation in normal nutrient conditions, we examined the effect of 705 µg/mL polyP on proliferation of mutants in SIH and HL5 media. In SIH, compared to WT cells, polyP had a reduced ability to inhibit the proliferation of *grlD*¯ and *lst8*¯ cells, and in HL5, polyP had a reduced ability to inhibit the proliferation of *grlD*¯, *adcB*¯, and *lst8*¯ cells (Fig. 4). Together, this indicates that some but not all polyP potentiation of bacterial survival signal transduction pathway components also mediate polyP proliferation inhibition, suggesting a partially shared pathway.

## DISCUSSION

**Fig 5:**
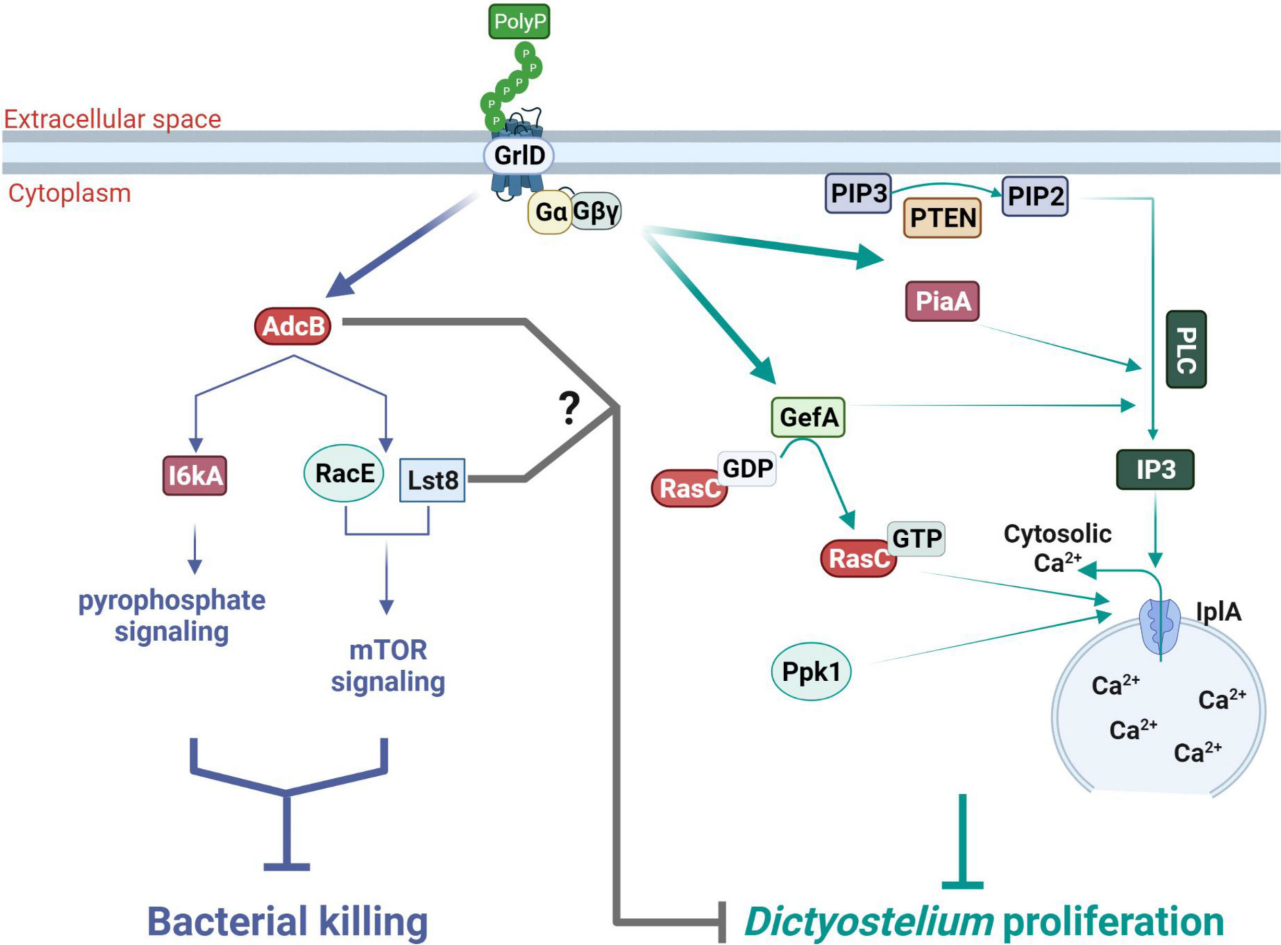
Hypothesized polyP signal transduction pathways that inhibit the killing of bacteria in *D. discoideum* and pathways that inhibit cell proliferation. PolyP binds to the GrlD receptor, and the polyP signal is relayed downstream of GrlD by the arrestin-like protein AdcB, inositol hexakisphosphate kinase A (I6kA), the Rho GTPase RacE, and the TOR component Lst8 to inhibit the killing of ingested bacteria. AdcB and Lst8 also help polyP to inhibit *D. discoideum* proliferation. To inhibit proliferation, polyP signaling downstream of GrlD receptor is also mediated by Gαs and Gβγ, PTEN, and PLC, which cleaves PIP2 to IP3 and DAG. When IP3 binds to the IP3 receptor IplA, Ca^2+^ is released into the cytosol. The increase of IP3 and cytosolic Ca^2+^ levels by polyP depends on PTEN, PLC, and IplA. GefA converts GDP-bound RasC to GTP-bound RasC. Both GefA and PiaA are essential for the upregulation of IP3 levels by polyP, while RasC and Ppk1 are necessary for the increase of cytosolic Ca^2+^ levels. The proposed pathway diagram was created using BioRender.com.

In this report, we found that to inhibit the killing of ingested bacteria in *D. discoideum*, polyP requires, in addition to the G protein-coupled receptor GrlD (16, 40), AdcB, I6kA, RacE, and Lst8. PolyP also appears to use AdcB and Lst8, but not I6kA and RacE, to inhibit proliferation. However, to inhibit killing of ingested bacteria, polyP does not require other components of the signal transduction pathway that it uses to inhibit cell proliferation such as Gβ, RasC, PakD, PiaA, PTEN, PLC, IplA, Ppk1, PiaA, and PkaC (23, 25), suggesting that polyP uses partially overlapping signal transduction pathways to inhibit proliferation and the killing of ingested bacteria (Fig. 5).

Although polyP requires the G protein subunit Gβ to inhibit proliferation in 25% HL5 (25), polyP does not require Gα3 and Gβ to potentiate the survival of ingested bacteria in *D. discoideum*, but rather uses the arrestin-like protein AdcB downstream of GrlD. Arrestins are scaffolding proteins that deactivate a G protein-coupled receptor by binding to the cytoplasmic domain of the receptor, which induces a conformational change that allows the release of the G proteins subunits bound to the receptor (30, 31). Therefore, polyP appears to activate different pathways immediately downstream of GrlD to inhibit proliferation and the killing of ingested bacteria.

Eukaryotic cells including *D. discoideum* utilize autophagy for intracellular degradation of cytoplasm and organelles (58, 63 – 67). In addition to preventing phagosome-lysosome fusion, pathogens such as *M. tuberculosis* and *Salmonella typhimurium* break out of phagosomes and enter the cytosol (68). The eukaryotic cell then degrades the disrupted phagosome and attempts to kill the pathogen in the cytosol by autophagy (68 – 70). *S. typhimurium*, which requires polyphosphate kinase to prevent their killing in human macrophages (71), show increased survival in *D. discoideum* cells lacking the autophagy proteins Atg6 and Atg7 (65). However, polyP does not need Atg6 and Atg7 to increase the survival of *E. coli* in *D. discoideum* cells. Assuming that *E. coli* are unable to break out of the phagosome*,* this indicates that polyP may not require autophagy machinery to potentiate the survival of ingested bacteria in phagosomes, and that autophagy is a second line of defense against pathogens that do escape the phagosome.

The high polyP concentrations that inhibit proliferation of *D. discoideum* cells also inhibit proteasome activity in *D. discoideum* and human leukemia cell lines (40), and inhibition of proteasome activity has been suggested as a potential therapeutic for cancer (72). The low polyP concentration (15 µg/mL) that does not inhibit proliferation but potentiates survival of bacteria inhibits proteasome activity in WT cells but not in *grlD^−^*, *adcB^−^ i6kA^−^, lst8^−^,* or *racE^−^* cells. This indicates that components of the polyP pathway which inhibit the killing of ingested bacteria also inhibit proteasome activity. One possibility is that this pathway allows some *D. discoideum* cells to sense the relatively low concentrations of extracellular polyP, signaling that they are near each other and will eventually overgrow their food supply and should begin to conserve energy by storing nutrients (not killing some of ingested bacteria) and decreasing protein degradation.

In this report, we found that polyP inhibits killing of ingested bacteria in *D. discoideum* cells using signal transduction pathway components and mechanisms that have orthologs in human cells. PolyP from ingested pathogenic bacteria inhibits their killing in human macrophages (16). An intriguing possibility is that macrophages have a pathway similar to the *Dictyostelium* pathway to sense polyP, and that blocking this pathway could induce macrophages to kill internalized pathogens such as *M. tuberculosis*.

### Contact for reagent and resource sharing

Further information and requests for reagents may be directed to, and will be fulfilled by, the authors Ramesh Rijal (rijalramesh@tamu.edu) and Richard Gomer (rgomer@tamu.edu).

## MATERIALS AND METHODS

### *D. discoideum* cell culture

*D. discoideum* strains were obtained from the *Dictyostelium* Stock Center (73) and were Ax2 (DBS0237699), Ax3 (DBS0235542), KAx3 (DBS0266758), DH1 (DBS0235700) , JH8 (DBS0236454), JH10 (DBS0236449), and HPS400 (DBS0236312), *adcB*¯ (DBS0350443)*, adcC*¯ (DBS0350646), *adcB*¯*/adcC*¯ (DBS0350445), *atg6*¯ (DBS0236344), *atg7*¯ (DBS0236372), *cnrN*¯ (DBS0302655), *cnxA*¯ (DBS0236189), *dagA*¯ (DBS0235559), *dymA*¯ (DBS0347874), *grlD*¯ (DBS0350227)*, gβ*¯ (DBS0236531), *gα3*¯ (DBS0235986)*, iplA*¯ (DBS0236260), *i6kA*¯ (DBS0236426), *i6kA*¯*/i6kA* (23), *lst8*¯ (DBS0236517)*, pakD*¯ (DBS0350281), *piaA*¯ (DBS0349879) *, pikA*¯ (DBS0350197), *pikB*¯ (DBS0350198), *pipkinA*¯ (DBS0236779), *pkaC*¯ (DBS0236783)*, pkbA*¯ (DBS0349876), *pkbA*¯/*pkgB*¯ (DBS0236785), *plC*¯ (DBS0267124), *ppk1*¯ (DBS0350686), *pten*¯ (DBS0236830), *racC*¯ (DBS0350272), *racE*¯ (DBS0235413), *racF1*¯ (DBS0351505), *racG*¯ (DBS0236849), *racH*¯ (DBS0236850), *rasC*¯ (DBS0236853), *rasG*¯ (DBS0236862), *rasC*¯*/rasG*¯ (DBS0236858), *scrA*¯ (DBS0236926) and *wasA*¯ (*wasA*¯ strain was a kind gift from Robert Insall, Beatson Institute for Cancer Research, Glasgow, UK) (2, 43, 46, 52, 53, 73 – 104). *D. discoideum* cell cultures were maintained at 21°C on lawns *Escherichia coli* B/R20 (Dictyostelium Stock Center) on SM/5 agar plates (105) and in type 353003 100 mm tissue culture dishes (Corning, Durham, NC) in 10 mL of HL5 medium or SIH defined minimal medium (Formedium, Norfolk, England). HL5 or SIH containing 100 µg/mL dihydrostreptomycin and 100 µg/mL ampicillin was used to kill *E. coli* in *D. discoideum* cultures obtained from SM/5 agar (105). Cells encoding a selectable marker were grown under selection with appropriate antibiotics and supplements (5 µg/mL blasticidin, 5 µg/mL neomycin sulfate, 100 µg/mL thymidine, and/or 20 µg/mL uracil). All experiments in Fig. 1 and 2 used cells from at least two different frozen stocks for each strain.

### Polyphosphate preparation

Sodium polyphosphate of average chain length of 45 monomers (16) (Cat#S0169, Spectrum, New Brunswick, NJ) was used for all assays. The sodium polyphosphate stock was prepared in PBM buffer [20 mM KH_2_PO_4_, 1 mM MgCl_2_, 0.01 mM CaCl_2_, pH 6.5 (23)] to a concentration of 70.5 mg/mL or 1.5 mg/mL, the pH was checked, and this stock was diluted 100× in cultures to make 705 µg/mL or 15 µg/mL polyP.

### Bacterial survival assay and phagocytosis

*E. coli* K-12 survival in *D. discoideum* was performed as previously described (16). *D. discoideum* cells were plated in type 353047 24-well plates (Corning) with 1 mL of cells at 10^6^ cell/mL in each well. After 30 minutes, polyP from a 1.5 mg/mL stock was added to a final concentration of 15 µg/mL, or an equal volume of PBM was added, and mixed gently using a pipette. *E. coli* K-12 bacteria were washed twice in PBM buffer by centrifugation at 12,000 × *g* for 2 minutes, followed by resuspension in PBM buffer. The OD_600_ was measured and bacteria were diluted in PBM to an OD_600_ of 0.1. *E. coli* K-12 (50 µL) was added to *D. discoideum* cells and incubated for 2 hours. To remove uningested extracellular bacteria, *D. discoideum* cells were gently washed with SIH. Any remaining bacteria that were not ingested were eliminated by adding gentamicin (Sigma) to a final concentration of 200 µg/mL. After 2 hours, the cells were washed again to remove gentamicin. At 4 and 48 hours after plating, cells were washed by centrifugation of plates at 500 × *g* for 3 minutes, removing the media, resuspending cells in 200 µL PBM, and 20 µL of the cell suspension was taken for *D. discoideum* cells count, and 150 µL of the cell suspension was mixed with 1.5% of Triton X-100 (Alfa Aesar), and then lysed by gently pipetting at room temperature. For the cell counts, 10 µL of cells was mixed with 10 µL of 0.4% Trypan blue (Cat#11618, Kodak, Rochester, NY), and after 30 seconds a TC20 automated cell counter (Bio-Rad, Hercules, CA, USA) was used to count live (unstained) and dead (stained) *Dictyostelium* cells. The Triton lysates were plated on LB agar for *E. coli* K-12 growth and incubated at 37°C. For cultures with polyP, PolyP was present in all incubation steps prior to the Triton lysis step. After 24 hours, colonies of *E. coli* K-12 were visible. The bacterial colonies were counted, and the viable bacteria within *D. discoideum* cells were calculated as cfu/10^6^ *D. discoideum* cells.

Phagocytic index is a measurement of the uptake of particles by phagocytes (106). Fluorescence microscopy was used to visualize ingested Alexa 594-labeled Zymosan-A yeast BioParticles (Cat#Z23374, Thermo Fisher Scientific) in *D. discoideum* as described in (16). Briefly, *D. discoideum* cells were seeded in type 353219 96-well, black/clear, tissue-culture-treated plates (Corning). After 30 minutes, polyP added to the cells to a final concentration of 15 µg/mL and mixed by gentle pipetting. Zymosan bioparticles were resuspended in PBM buffer to a concentration of 0.5 mg/mL. Ten microliters of bioparticles was added to the cells and mixed by gentle pipetting, and the plates were spun down at 500 × *g* for 3 minutes. After 1 hour, images of *D. discoideum* cells were taken with a 40× objective on a Nikon Eclipse Ti2 (Nikon), and we used the Richardson-Lucy algorithm (107) for deconvolution of images in NIS-Elements AR software. The number of bioparticles ingested per cell per hour was calculated as a mean number of ingested bioparticles per phagocytosing *D. discoideum* cell multiplied by the percentage of *D. discoideum* cells engaged in phagocytosis.

### Genotype verification

The genotype of *D. discoideum* strains that were insensitive to polyP (*grlD*¯, *adcB*¯, *i6kA*¯, *lst8*¯, and *racE*¯) were verified by PCR as previously described (53) using the specific primer pairs listed in Table 2.

**TABLE 2:** Oligonucleotides for genotyping of polyP insensitive mutants by PCR

cDNA	5’−3’ Forward primer	5’−3’ Reverse primer
***grlD***	ATGAAAATTAATTCATTTTT	TTAATTATCACCATCATTATTTTCTTC
***adcB***	ATGGATAACAGAGGATTAAG	CTAATTATTTAATTTAAG
***i6kA***	ATGCACATATTTTATTTAGTAAACTCG	GTTTGTATTTATGACTGTATTTTGTTG
***racE***	ATGTCAGAAGATCAAGGTTCAGG	TTAAAGTATAATACAACCAG
***lst8***	ATGCCAGGTATTATATTGGC	TTATCTTGGTAAATCATTTAAAGC

### Immunoblotting

One milliliter of 10^6^ *D. discoideum* cells in SIH was seeded in a type 353047 24-well tissue culture plate (Corning), and either 15 µg/mL polyP from a 15 mg/mL stock in PBM (20 mM KH_2_PO_4_, 1 mM MgCl_2_, 0.01 mM CaCl_2_, pH adjusted to 6.1 with KOH) or an equivalent volume of PBM was added. At 4 or 48 hours, the 24-well plate was centrifuged at 500 × *g* for 3 minutes, and the supernatant was replaced by 1 mL of PBM. This step was repeated once. The 24-well plate was centrifuged at 500 × *g* for 3 minutes, the supernatant was discarded, and cells at the bottom of each well were lysed with 100 µL of 1× SDS sample buffer. The lysates were collected, and Western blots and Coomassie staining of gels was performed as previously described (53). Western blots were stained with 1:1000 diluted mouse monoclonal anti-ubiquitin antibodies (Cat# 3936T; Cell Signaling Technology) to detect ubiquitinated proteins.

### Proteasome activity assay

One hundred microliter of *D. discoideum* cells in SIH at 10^6^ cells/mL was seeded in type 353219, 96-well, black/clear, tissue-culture-treated, glass-bottom plates (Corning), spun down at 500 × *g* for 3 minutes, the medium was changed to SIH or SIH containing 15 µg/mL polyP, and the 96-well plate was incubated in a Tupperware container with wet paper towels (for humidity) for 48 hours. Proteasome activity was measured using a Proteasome Activity Kit (Cat#MAK172, Sigma, St Louis, MO) following the manufacturer’s instructions.

### Proliferation inhibition

Proliferation of *D. discoideum* strains in the presence or absence of 705 µg/mL polyP was measured as previously described (23). Briefly, cells grown in HL5 or SIH (as described above) were diluted to 5 × 10^5^ cells/mL in 1 mL of HL5 or SIH, incubated in the presence or absence of 705 µg/mL polyP and the cell density was measured after 24 hours with a hemocytometer. For each strain, the increase in cell density in the presence of polyP was calculated as a percentage of the increase in cell density in the absence of polyP.

### Statistical analysis

Statistical analyses were performed using Prism 9 (GraphPad, San Diego, CA) and the tests indicated in the figure legends. A *P* < 0.05 was considered to be significant.

## Data Availability

The article and its associated supplementary data contain all the necessary data to support the results. Further information may be directed to, and will be fulfilled by, the authors Ramesh Rijal (rijalramesh@tamu.edu) and Richard Gomer (rgomer@tamu.edu).

## SUPPLEMENTAL MATERIAL

Supplemental Figures mbio.01939-23-s0001.docx Fig. S1-S3.

ASM does not own the copyrights to Supplemental Material that may be linked to, or accessed through, an article. The authors have granted ASM a non-exclusive, world-wide license to publish the Supplemental Material files. Please contact the corresponding author directly for reuse.
