# Supplementary material for: Polyphosphate uses mTOR, pyrophosphate, and Rho GTPase components to potentiate bacterial survival in Dictyostelium
Source: mBio. 2023 Sep 27;14(5):e01939-23. doi: 10.1128/mbio.01939-23 (PMC10653871; doi:10.1128/mbio.01939-23)
Supplement: Supplemental Figures — Fig. S1-S3. [file mbio.01939-23-s0001.docx]

Polyphosphate uses mTOR, pyrophosphate, and Rho GTPase components to potentiate bacterial survival in *Dictyostelium*

Ryan J. Rahman, Ramesh Rijal#, Shiyu Jing, Te-An Chen, Issam Ismail, and Richard H. Gomer#

Department of Biology, Texas A&M University, College Station, Texas, USA

Running Head: Bacterial survival mechanism in a phagosome

#Address correspondence to Richard H. Gomer, rgomer@tamu.edu or Ramesh Rijal, rijalramesh@tamu.edu

Department of Biology, Texas A&M University, ILSB 301 Old Main Drive

College Station, Texas, USA

**
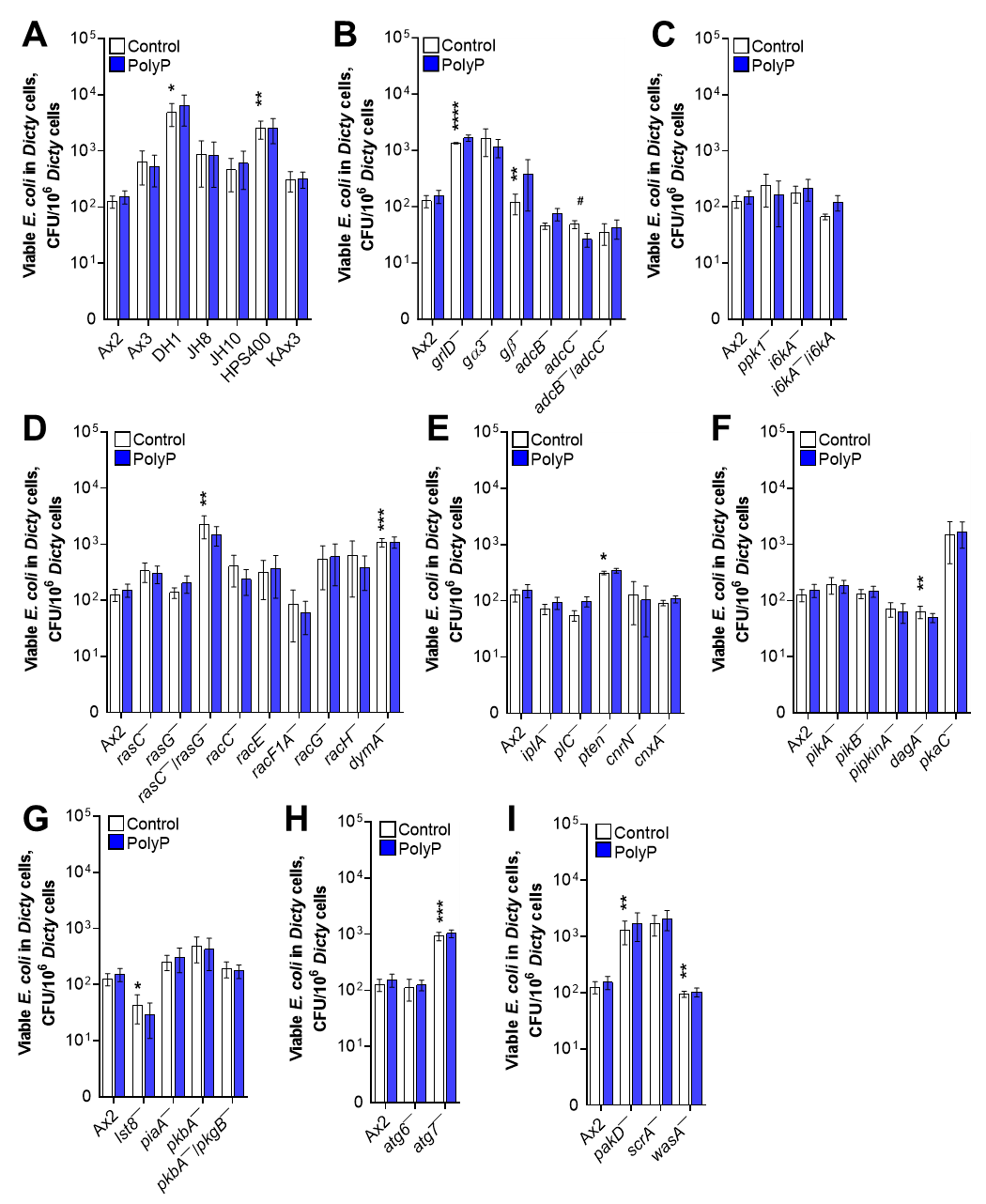
Supplementary Figures**

**Figure S1: PolyP does not potentiate the survival of ingested *E. coli* in most *D. discoideum* strains at 4 hours.** (A-I) *D. discoideum* (Dicty) cells were incubated with *E. coli*, uningested *E. coli* were removed, and the number of viable ingested *E. coli* per 10^6^ *D. discoideum* cells in the absence (Control) or the presence of added polyphosphate (PolyP) was determined at 4 hours. Values are mean ± SEM from 5 independent experiments for each mutant/parental strain and 16 independent experiments for Ax2 wild type. * p < 0.05, ** p < 0.01, *** p < 0.001, and **** p < 0.0001 by 2-tailed Mann-Whitney test comparing the indicated mutant to its parental strain (Table 1), or the indicated parental strain to Ax2, in the absence of added polyP. # p < 0.05 by 2-tailed Mann-Whitney test comparing control to polyP for the indicated strain.


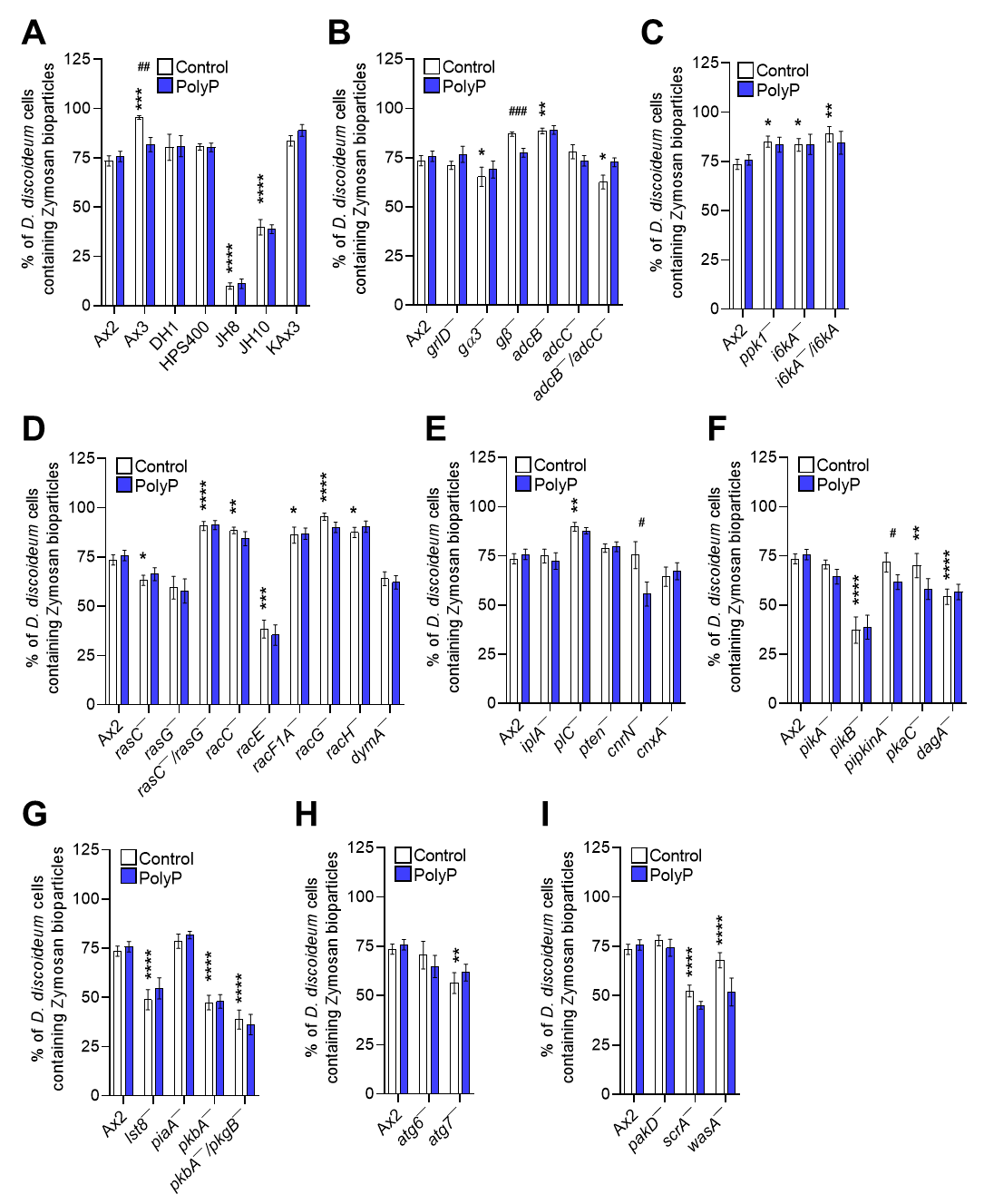
**Figure S2:** **PolyP has negligible effect on the uptake of Zymosan A bioparticles in most strains.** (A-I) The percentage of *D. discoideum* cells with ingested Zymosan A bioparticles after 60 minutes in the presence or absence of 15 µg/ml polyP was determined. Values are mean ± SEM from 3 independent experiments. * p < 0.05, ** p < 0.01, *** p < 0.001 and **** p < 0.0001 comparing the indicated mutant to its parental strain, or the indicated parental strain to Ax2, in the absence of added polyP by 2-tailed Mann-Whitney test. # p < 0.05 and ### p <0.001 comparing control and polyP for the indicated strain by 2-tailed Mann-Whitney test.

**
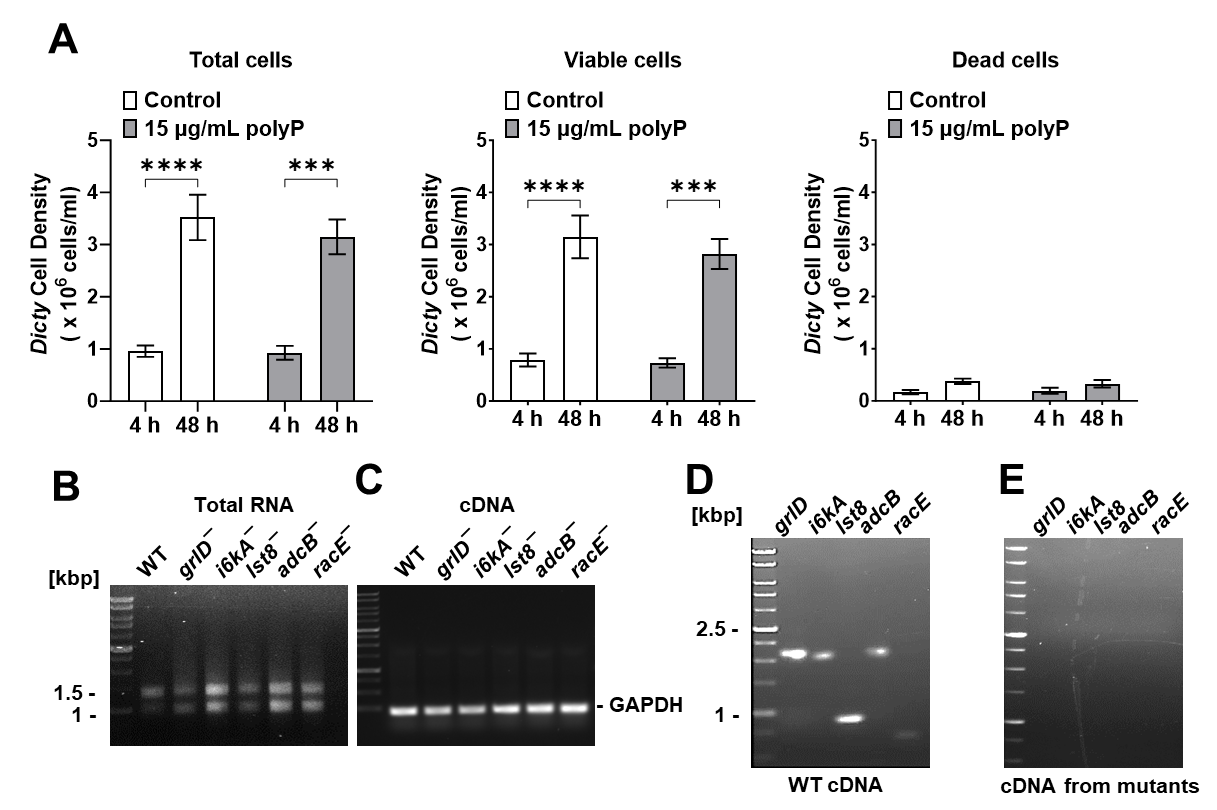
Figure S3:** ***Dictyostelium* viability and genotyping of mutants.** (A) *D. discoideum* (Dicty) cells were incubated with *E. coli*, uningested *E. coli* were removed, and the density of *D. discoideum* cells with ingested *E. coli* in the absence (Control) or the presence of added polyphosphate (PolyP) was determined at 4 and 48 hours. Trypan blue staining was used to determine live and dead cell densities. Values are mean ± SEM, n = 5. *** p < 0.001, **** p < 0.0001 by Holm-Šídák's multiple comparisons test (2way ANOVA). (B) Total RNA was isolated from WT and the indicated mutant strains and separated by agarose gel electrophoresis. Molecular masses of DNA markers in kbp are at left; the two prominent bands are ribosomal RNAs (upper band is 26S and lower band is 17S), where S indicates Svedberg sedimentation coefficient. (C) PCR was done using gpdA (GAPDH) specific primer pairs as a control for the presence of cDNA in the indicated strains. Molecular masses in kbp are at left. (D-E) PCR was done as in B using the gene-specific primer pairs (Table 2) using WT cDNA (D) or the respective mutant’s cDNA (E).
